# Acute Effects of Self-Correction on Spine Deviation and Balance in Adolescent Girls with Idiopathic Scoliosis

**DOI:** 10.3390/s22051883

**Published:** 2022-02-28

**Authors:** Luca Marin, Nicola Lovecchio, Luisella Pedrotti, Federica Manzoni, Massimiliano Febbi, Ilaria Albanese, Pamela Patanè, Vittoria Carnevale Pellino, Matteo Vandoni

**Affiliations:** 1 Laboratory of Adapted Motor Activity (LAMA), Department of Public Health, Experimental Medicine and Forensic Science, University of Pavia, 27100 Pavia, Italy; vittoria.carnevalepellino@unipv.it (V.C.P.); matteo.vandoni@unipv.it (M.V.); 2 Laboratory for Rehabilitation Medicine and Sport (LARMS), 00133 Rome, Italy; massimilianofebbi@gmail.com (M.F.); ilaria.albanese@gmail.com (I.A.); pamela.patane01@universitadipavia.it (P.P.); 3 Department of Rehabilitation, Città di Pavia Hospital, 27100 Pavia, Italy; 4 Department of Research, ASOMI College of Sciences, 2080 Marsa, Malta; 5 Department of Human and Social Science, University of Bergamo, 24127 Bergamo, Italy; nicola.lovecchio@unibg.it; 6 Orthopedics Unit, Department of Clinical Surgical Sciences, Diagnostic and Pediatrics, University of Pavia, 27100 Pavia, Italy; luisella.pedrotti@unipv.it; 7 Department of Pediatric Orthopedics, Città di Pavia Hospital, 27100 Pavia, Italy; 8 Epidemiological Observatory Unit, Health Protection Agency, 27100 Pavia, Italy; federica.manzoni@unipv.it; 9 Department of Industrial Engineering, University of Tor Vergata, 00133 Rome, Italy

**Keywords:** adolescent girls with idiopathic scoliosis, self-correction movement, balance, postural control and spine movement

## Abstract

Background: Adolescent idiopathic scoliosis (AIS) is a three-dimensional deformity of spine and trunk with a higher incidence in girls. AIS alters and reduces postural control and balance. Self-correction movement (SCM) is a well-known non-invasive approach to ameliorate spine curve in AIS subjects. We aimed to evaluate the effects of SCM on the spine and on the balance of adolescents with AIS with a new non-invasive instrumentation. Methods: A total of 38 girls with AIS were recruited. To evaluate the acute effects of SCM and the oscillations of center of pressure (COP), we used LiDAR technology combined with a stabilometric platform to evaluate both changes in spinal curves and balance at the same time. Two tests were carried out simultaneously using the two instruments: before the execution of SCM, in the spontaneously assumed position of each subject (SP) and after the execution of SCM, during the achieved position (SC). Sway area, COP medio-lateral and antero-posterior directions, eccentricity of the ellipse and vertebral lateral deviation were recorded. The two conditions were compared with a Wilcoxon signed-rank test. Results: In general, all measures showed lower values in SC condition (*p* < 0.05), except the variation along the Y axis. Conclusions: Thanks to objective measured data, the therapists observed real-time changes during the performance of SCM, appreciating its efficacy on curve correction.

## 1. Introduction

Adolescent idiopathic scoliosis (AIS) is defined as a three-dimensional deformity of spine and trunk with a predominance of sideways deviation on the other movement planes and with higher incidence in girls than in boys [1]. The AIS etiology is multifactorial in 80% of cases (genetic predisposition, connective tissue and skeletal abnormalities, muscular and neurological disorders during growth) and defined as idiopathic [2]. An early onset of AIS is crucial as a predictor of the prognosis aggravation and to fully meet the primary goal of specific treatments: reduction in progression and improvement of health-related quality of life in adulthood [2,3]. To this point, there are different strategies to manage AIS, whereas the most widespread and preferred approach is based on specific physiotherapy exercises able to limit the progression of the curve [4,5] with non-invasive procedures.

There are several methods and typologies of exercises for treating AIS. Most are based on strengthening and stretching the affected muscle groups. Others, as suggested by the 2016 SOSORT guidelines, are based on self-correction along the three planes [6]. Thus, the self-correction movements (SCM) are characterized by accurate and well-targeted movements deputed to reduce scoliotic curve and require the assistance of a professionist. Usually, SCM is performed in three planes: in the frontal plane the correction is performed through the shift of the convexity curve towards the concavity zone, in the horizontals through localized de-rotation of the scoliotic vertebrae and, finally, in the sagittal plane reaching the physiological dorsal kyphosis. These analytic exercises need the assistance of a physiotherapist to provide the correct cues, to increase body perception and, in turn, autonomy during SCM within gym practice or daily-living activities [7,8]. In this framework, it is important to highlight that scoliosis alters and reduces postural control and personal motor control because of the action of an external stimulus [9]. Poor body segmental alignment caused by AIS has been associated with increased lateral displacement of the body center of mass (COM), affecting dynamic balance during walking. A previous study by Ceballos Laita et al. 2018 reported that better postural control could be related to the capacity to maintain SCM for a longer period [10]. Moreover, a study by Monticone et al. (2014) showed that a program with active self-correcting exercises was useful to reduce the course of spinal deformity and improve quality of life in adolescents with mild AIS [11].

In particular, the most used method to assess postural control [12,13] is the measurement of the center of pressure (COP) [14,15], which is the center of distribution of pressure on the ground that estimates the oscillation of the COM during body arrangement [16]. The magnitude of COP displacement is also used as an index to evaluate the effects of self-elongation movements during exercise. In fact, during SCM, the sway of the COP shows a balanced trend along two directions: antero-posterior and medio-lateral [17].

In pediatrics, the gold standard for the initial diagnosis and longitudinal surveillance is the two-dimensional (2D) posterior-anterior full-length spine radiography [18], but frequent radiological assessments during growth could lead to long-term adverse effects [19,20]. Alternative radiation-free measurement methods are increasingly used to determine the shape of the spine, reducing the possible health risks caused by repeated X-ray exposure [21]. One of these methods is based on stereophotogrammetric that [22,23] needs darkness and is difficult to reproduce in gym practice or in a standardized clinical setting. Nowadays, a new technological system can reproduce spine images on a monitor using an infrared camera (ToF cameras), capturing the image as the difference between the projected image and the acquired image without crosstalk signal from the environmental light [24]. This scanning method is markerless, radiation-free, non-invasive [25] and does not require room darkness for the images’ acquisition, allowing multiple captures of spine images with a three-dimensional model of the spine and pelvis in different angles [26]. In light of this, Betsch et al. emphasized that the functional analysis of real time posture can be performed under dynamic conditions using the rasteosterography (RS) technique [27,28]; dynamic analysis of a scoliotic spine should be implemented to monitor the trends of curves during every exercise sessions [27,29].

For these reasons, with the present work, we aimed to evaluate the acute effects of the SCM on the spine and on the balance of adolescents with AIS. These subjects have less stability than healthy people caused by the variations of the normality of the spine and the altered head position [8,29] that, combined, elicit an altered dynamic proprioception [9].

## 2. Materials and Methods

### 2.1. Study Design and Participants

We conducted an observational study. A total of 38 girls with AIS were recruited from the Orthopedics Department of the “Città di Pavia”—University Hospital of Pavia during the weekly physiotherapy routine session. The inclusion criteria were the age between 12 to 17 years old, the ability to perform self-elongation exercises and the scoliotic curve with a Cobb angle lower than 40°. The exclusion criteria were the presence of a secondary scoliotic curve, orthopedic injuries in the last six months, the presence of a cardiovascular or metabolic disease such as obesity, the presence of a disease that that could lead to neurological or vestibular impairments and the engagement in more than three days per week of sport activity practice. The physiotherapists explained all the procedures to the parents and the adolescents before engagement in the study protocol and were advised that they could withdraw from the study at any moment. Parents or legal guardians and adolescents gave their verbal and written informed consent. All the procedures were in accordance with the Declaration of Helsinki (1975) as revised in 2013 [30] and were preventively approved by the clinic ethics committee (Area Vasta Pavia Ethical Committee Protocol code 20180036031) and registered on ClinicalTrials.gov (NCT04268082).

### 2.2. Anthropometric Measurements

Anthropometric measurements were performed as described elsewhere [17]. Body mass was measured barefoot and in light clothing, standing upright in the center of the scale platform (Seca, Hamburg, Germany) with hands along the trunk. Stature was measured using a Harpenden stadiometer (Holtain Ltd., Cross-Well, UK) with a fixed vertical backboard and an adjustable head piece. The measurement was taken with subjects in an upright position, without shoes, and the head in the Frankfort horizontal plane. Two measurements were taken for each parameter, and a third was obtained if a discrepancy of 500 g and 0.5 cm were noted between the initial measurements. The anthropometric parameters were then based on the average of the two closest measurements. Cobb angles [31] were measured according to specific guidelines [17] while the Risser sign [32] was determined from an X-ray image of the pelvis and then compared with references [17].

### 2.3. Instruments

**Figure 1:**
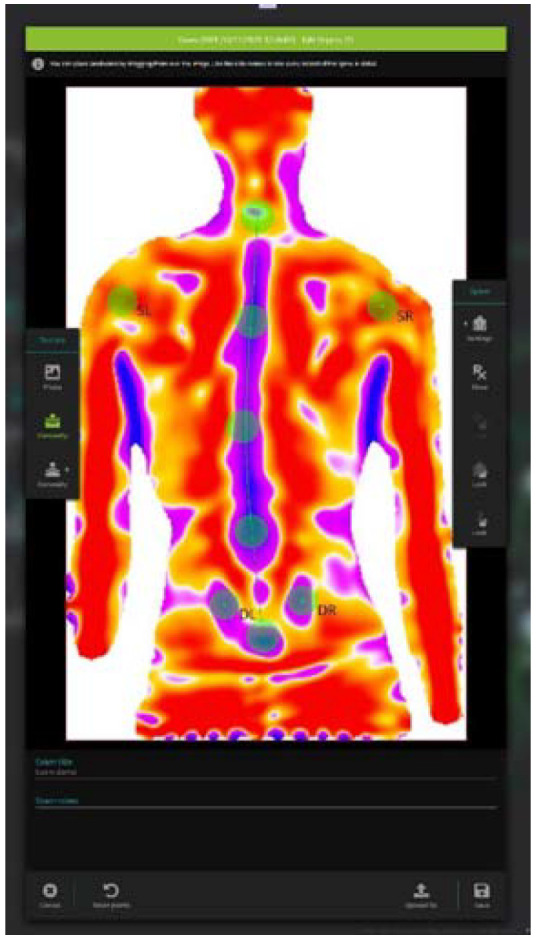
The reconstruction of the back image acquired and showed by the Spine 3D instrument on the panel.

To evaluate the acute effects of SCM on spine curves, we used light detection and ranging technology (LiDAR) called Spine 3D (Sensormedica, Guidonia Montecelio, Rome, Italy): an innovative and non-invasive three-dimensional optoelectronic detection system (Kinect) that allows an accurate assessment of vertebrae alignment. The spine 3D system is composed of a single vertical aluminum panel of 165 × 63 × 76 cm dimensions with a resolution of 1920 × 1080 pixels and 30 fps frame rate of acquisition. The system uses infrared cameras called “Time of Flight” (ToF) that allow the measurement of the reflection of the light (camera-subject-camera) without the necessity of the use of a dark room for the acquisition. The internal software, through an infrared light beam, captures the image of the back, records the difference between the projected image and the acquired one that is showed on a panel. The captured image is shown in Figure 1. The data obtained from the surface irregularities, automatically identified by the instrument such as the prominent vertebra, the right and the left shoulder and the right and the left lumbar dimple, were mathematically analyzed to track the morphology of the column with a three-dimensional rendering (resolution of 1 mm).

To evaluate the oscillation of COP during the acquisition (that correspond to a self-elongation exercise), a stabilometric platform with resistive 24 k gold-coated sensors was used (FreeMed^®^, Sensormedica, Rome, Italy). Dimensions were 80 × 50 cm and the use of a sampling frequency of up to 400 Hz guaranteed a high level of accuracy during the stance phase (Intra Class Correlation between 0.80 and 0.83); [13]. All the data were processed by a computer using the FreeStep© software (Sensormedica, Rome, Italy) that is able to elaborate different acquisitions into a large database. Moreover, the software, in real time (i.e., during acquisitions), shows the pressure level of a single foot and then the total load.

### 2.4. Procedures

**Figure 2:**
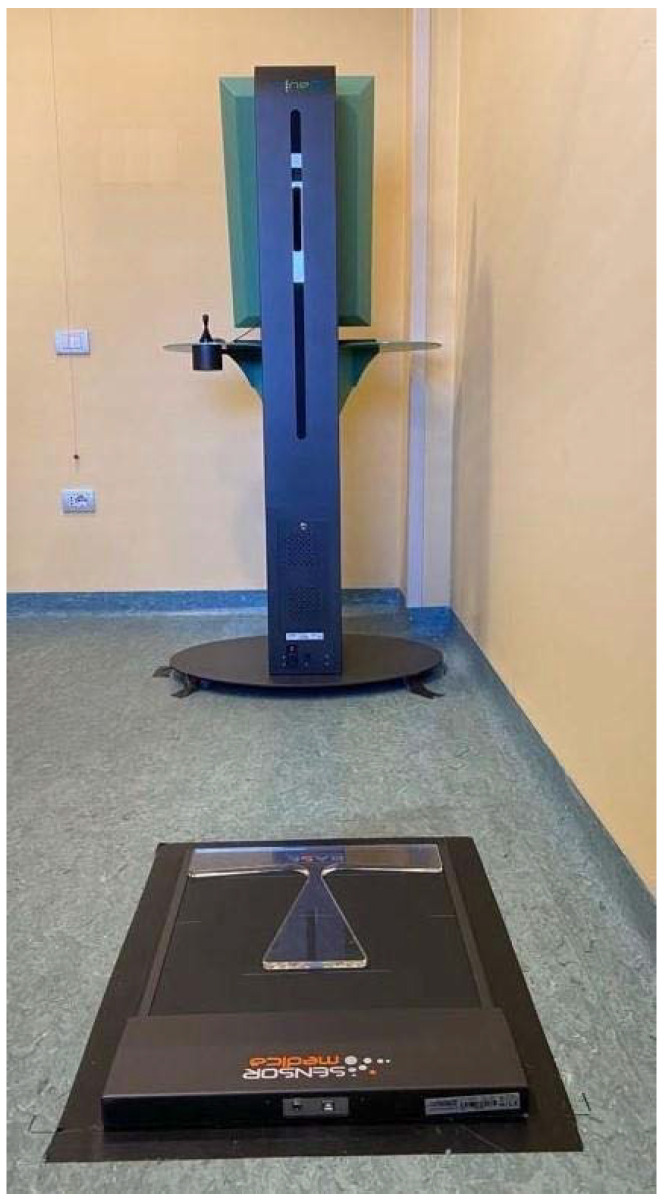
The clinical setting for the study protocol with the two instruments in the correct position for the acquisitions.

All the measurements were assessed in the afternoon (4–5:00 or 5–6:00 p.m.) in the same clinical setting; specifically, in a room in a clinical context with a constant temperature of 21 °C. The clinical setting is shown in Figure 2. Participants stood barefoot on the stabilometric platform, in a quiet erect stance, with the uncovered back facing the Spine 3D that was positioned at 110 cm from the subjects. Two tests were carried out using the two instruments simultaneously: before the execution of SCM, in the spontaneously assumed position of each subject (SP) and after the execution of SCM, during the position achieved (SC). Each test lasted ten seconds keeping the feet position and were performed in a quiet erect stance.

SCM works in the three planes of the space: in the frontal plane the correction is performed through the shift of the convexity curve towards the concavity zone, in the horizontals through a localized de-rotation of the scoliotic vertebrae and, finally, in the sagittal plane reaching the physiological dorsal kyphosis.

The baropodometric platform software provided sway area (SA; cm^2^); the COP medio-lateral and antero-posterior directions, respectively, defined sway-X (mm) and sway-Y (mm). Then, the eccentricity of the ellipse (EE) was extracted as an index of the symmetry of the COP displacement [16]. This value defines the shape skewness of an ellipse considering the length of the two axes. Usually, this value ranges between 0 and 1; where the zero represents an ellipse quasi-overlapping to a circle while in case of 1 the ellipse corresponds to a line. Finally, we evaluated the vertebral lateral deviation (VLD) using the RMS function applied to the distance between the vertebrae and the ideal line (defined as the whole vertebra alignment on the frontal plane) that corresponds to a value of 0. In fact, the instrument acquires the position of the vertebra spinous process and matches it with the Kendall ideal line. In particular, this index was calculated as the quadratic mean of the horizontal lateral deviation considering the center of the vertebrae.

### 2.5. Statistical and Data Analysis

All the quantitative variables are shown as median and IQR. We tested the normality using a Shapiro–Wilk test. To evaluate the differences between the two conditions (standing, SP and during the position achieved after SCM (SC)), data were matched and compared with a non-parametric approach through a Wilcoxon signed-rank test. To ensure the correct statistical power, we calculated the sample size of the study. To detect a 10% variation of subjects with a balanced distribution of the COP in the sample, a total of 36 patients guarantees a power of 81%, with an alpha error of 0.05. Considering a dropout rate of 5%, we decided to enroll at least 38 patients. The significance was set at a p-value less than 0.05 and eventually the effect size (ES) was used to verify the biological consistence of the differences. Statistical analyses were performed using the Jamovi project (2021): jamovi Version 1.6 for Mac, Sydney, Australia (Computer Software).

## 3. Results

A total of 36 girls with AIS completed the study because two subjects were excluded since they did not meet the inclusion criteria. Descriptive characteristics are shown in Table 1. In general, all the measures showed lower values in SC condition (*p* < 0.05), except for the variation along the Y axis (anterior-posterior direction). All the peculiar results are described in Table 2, whereas the most important modification regards the significant reduction in the VLD.

## 4. Discussion

AIS, among a plethora of disorders, alters posture with negative sequelae for balance control and, recently, physiotherapy treatments are assessed through a stabilometric evaluation [17,33,34]. At the same time, a new generation of instruments permits the evaluation of the scoliotic curve using ToF cameras without radiation and environmental light interferences. Thus, thanks to this improvement, the investigation of scoliotic curve during SCM can be performed more accurately. Consequentely, the benefits/correctness of SCM can be evaluated in acute condition and in an objective way and not only by ‘the view’ of the therapist [17].

Our study showed a reduction in VLD in SC position obtained by a better spine alignment in respect to SP. Thanks to the objectively acquired data, the therapist observed the real-time changes during the performance of SCM. Our results are in line with Dupuis et al. that found that SCM improve spine alignment (showed by LiDAR technology) reducing scoliotic curve amplitude [35] and, as recently verified [14], we report that SCM is also able to ameliorate the balance control (eccentricity close to 0 and reduction in SA) and the treatment of AIS without an invasive approach. The value of Sway-Y in the SC position did not decrease, probably because of the better control [8] in medio-lateral direction, which is the plane where the self-elongation occurred [36]. Furthermore, our results indicate that the EE index is significantly better in SC than in SP, highlighting the role of the SCM in postural control and the balance of adolescents with AIS. These findings are in agreement with other authors [17,37,38], who underlined the need to improve proprioception to generally increase postural control.

The SCM, if well supervised, is an effective and non-invasive approach to improve scoliosis in acute. The acute positive effects on spine alignment could contribute to increasing self-proprioception across the therapy sessions and consequently to bettering responses to the rehabilitation process. In particular, our study aimed to evaluate the possibility of using a new, accurate, non-invasive and relatively affordable instrument to measure the effect of SCM in real-time during clinical practice. In fact, these new technologies are able to measure minimal movements of body alignment with a direct repercussion on the management of the therapy program. We are conscious that this study had some limitations. Firstly, we enrolled only girls in the sample and our results can not be generalized to both genders. Secondly, we did not evaluate patients with more thoracic-lumbar curves, underlining the necessity to implement our observations in the worst cases of scoliosis. Thirdly, we are conscious that, despite the benefits of these technologies, the higher cost of them may not be in reach of all those interested in postural assessments. Finally, even if the therapist had knowledge of the physical activity level through the recorded anamnesis of the subjects, we did not evaluate the physical activity level of the enrolled subjects with a valid questionnaire. Furthermore, further studies are desirable to evaluate the biofeedback effect combined with new technologies in the treatment of AIS patients.

## 5. Conclusions

In light of these results, SCM can objectively contribute to ameliorating spine curve and to the treatment of AIS without an invasive approach. These new technologies help the frequent and real-time monitoring of the curve progression and the auto-correction of posture. Moreover, the therapist could ameliorate the methodology of exercise prescription with immediate and objective feedback to the patients. These treatment improvements could also be translated to a correction of posture during daily life [39]. In conclusion, we hope that the new technologies and their development could contribute to ameliorating the cure, treatment and quality of life of AIS patients.

## Figures and Tables

**Table 1:** Descriptive characteristics of the whole sample.

	Total Sample (*n* = 36)
Age (years)	14 (2.0; 13.0–15.0)
Body mass (kg)	52.2 (9.9; 45.6–55.5)
Stature (m)	1.60 (1.0; 1.5–1.6)
BMI (kg/m^2^)	19.5 (3.9; 18.0–21.9)
Cobb (°)	14 (9.0; 10.0–19.0)
Risser	3 (2.0; 2.0–4.0)

All values are shown as median (IQR; 25–75th percentiles).

**Table 2:** Sway and Spine 3D outcomes in the two different conditions.

		Median (IQR; 25–75th)	*p*-Value	ES
EE	SP	0.5 (0.4; 0.3–0.8)	0.002 *	0.6984
	SC	0.3 (0.3; 0.1–0.5)		
Sway X (mm)	SP	9.6 (5.2; 6.5–11.7)	0.050 *	0.4161
	SC	7.4 (3.3; 6.0–9.4)		
Sway Y (mm)	SP	8.3 (3.6; 6.6–10.2)	0.749	−0.0713
	SC	9.2 (3.4; 7.3–10.2)		
SA (cm^2^)	SP	95.6 (98.0; 50.5–148.6)	0.035 *	0.4483
	SC	59.4 (49.3; 37.9–87.2)		
VLD (mm)	SP	5.4 (4.0; 3.1–7.1)	0.020 *	0.4897
	SC	3.8 (2.7; 2.4–5.1)		

All values are shown as median (IQR; 25–75th percentiles). * Significance at *p* < 0.05.; EE = eccentricity of the ellipse; Sway X = medio-lateral direction; Sway Y = anterior-posterior direction; SP = standing position; SA = sway area; SC = self-correction position; VLD = vertebral lateral deviation.

## Data Availability

Data can be requested from the corresponding authors (L.M.) upon reasonable request.
